# Reliability of Stereophotogrammetry for Area Measurement in the Periocular Region

**DOI:** 10.1007/s00266-020-02091-5

**Published:** 2021-01-15

**Authors:** Jinhua Liu, Alexander C. Rokohl, Yongwei Guo, Senmao Li, Xiaoyi Hou, Wanlin Fan, Maxim Formuzal, Ming Lin, Ludwig M. Heindl

**Affiliations:** 1 Department of Ophthalmology, Faculty of Medicine and University Hospital Cologne, University of Cologne, Kerpener Strasse 62, 50937 Cologne, Germany; 2 Eye Center, Second Affiliated Hospital, School of Medicine, Zhejiang University, Hangzhou, Zhejiang China; 3 Department of Ophthalmology, Shanghai Ninth People’s Hospital, Shanghai Jiao Tong University School of Medicine, Shanghai, China; 4 Center for Integrated Oncology (CIO) Aachen-Bonn-Cologne-Duesseldorf, Cologne, Germany

**Keywords:** Three-dimensional stereophotography, Periocular region, Area measurements, Reliability

## Abstract

Three-dimensional (3D) stereophotography area measurements are essential for describing morphology in the periocular region. However, its reliability has not yet been sufficiently validated. The objective of this study was to evaluate the reliability of 3D stereophotogrammetric area measurements in the periocular region. Forty healthy volunteers had five flat paper objects placed at each of the seven periocular positions including the endocanthion and the upper medial, upper middle, upper lateral, lower medial, lower middle, and the lower lateral eyelid. Two series of photographic images were captured twice by the same investigator. Each image of the first series was measured twice by the same rater, while images of both series were measured once by a second rater. Differences between these measurements were calculated, and the intrarater, interrater, and intramethod reliability was evaluated for intraclass correlation coefficients (ICCs), mean absolute differences (MADs), technical errors of measurements (TEMs), relative errors of measurements (REMs), and relative TEM (rTEM). Our results showed that 21.2% of all ICCs were considered as excellent, 45.5% were good, 27.3% were moderate, and 6.1% were poor. The interrater ICC for the endocanthion location was 0.4% on a low level. MAD values for all objects were less than 0.3 mm^2^, all TEM were less than 1 mm^2^, the REM and rTEM were less than 2% for all objects, showing high reliability. 3D stereophotogrammetry is a highly reliable system for periocular area measurements and may be used in the clinical routine for planning oculoplastic surgeries and for evaluating changes in periocular morphology.

*Level of Evidence IV* This journal requires that authors assign a level of evidence to each article. For a full description of these Evidence-Based Medicine ratings, please refer to the Table of Contents or the online Instructions to Authors www.springer.com/00266.

## Introduction

Quantitative analysis of facial anthropometry plays a vital role in the monitoring of growth, plastic surgery design, postoperative effect evaluation, as well as the measurement of facial morphology and deformity [1]. Facial anthropometric measurements include calipers, two-dimensional photographs, and three-dimensional (3D) methods such as stereophotogrammetry [2]. Direct skin contact, continuous cooperation, and image distortion due to depth illusion deficiency are inevitable for both traditional direct or two-dimensional measurement methods, also, the accurate measurement of area and volume is unattainable for these two methods [3].

In recent years, the development of high-resolution camera technology has enabled stereophotogrammetry to play an increasingly important role in the description of facial morphologies [4]. Images can be acquired noninvasively, rapidly, and precisely without any radiation. The 3D Cartesian coordinate system (*x*, *y*, and *z*) of stereophotogrammetry enables the mathematical calculation of depth [5]. Based on the depth, measurements for area and volume become a unique advantage and provide a novel and accurate facial description method. Compared with traditional linear measurement (composed of two points) or angle measurement (composed of three points), area or volume-based-stereophotogrammetry application becomes more and more important in clinical practice [6–8]. However, volume measurement is claimed to be inaccurate according to the technical information and product introduction provided by the manufacturer of the 3D stereophotogrammetry Vectra-M3 system (https://www.canfieldsci.com/imaging-systems/vectra-m3-3d-imaging-system/), and some researcher also reported that the accuracy of volume measurement by 3D stereophotogrammetry is not satisfactory when measuring delicate parts such as periocular tissues [5].

Areal measurement consists of numerous measurement points in the target area and are able to provide more information. The accuracy and reliability of stereophotography in the linear distance and angle measurements have been verified in previous studies [3, 9–13]. Unfortunately, the reliability of area measurements in the periocular region (the surface anatomy of the periorbital region which includes brow, forehead, upper eyelid, lower eyelid, and midface) has not been fully verified. As stereographic areal measurement holds a promising clinical application in the periocular region (such as evaluation for eyelid edema, eyelid scar or eyelid lesion), it is necessary to verify its feasibility (accuracy and reliability) before extensive clinical application.

This study aimed to demonstrate the reliability of stereophotography for areal measurements, with different measuring targets in different periocular locations, including the interrater reliability, intrarater reliability, and intramethod reliability of the VECTRA M3 system.

## Materials and methods

### Participants

For this study, 40 healthy volunteers under 40 years old were recruited at the Department of Ophthalmology of XXX University. Volunteers with facial deformities or abnormalities, facial trauma, and dermal diseases were excluded. Written informed consent was obtained from all participants before the photographs were taken. This study complied with the Declaration of Helsinki and its later amendments. Approval was obtained from the local institutional ethics committee (Number: 17-199).

## Objects

**Fig. 1:**
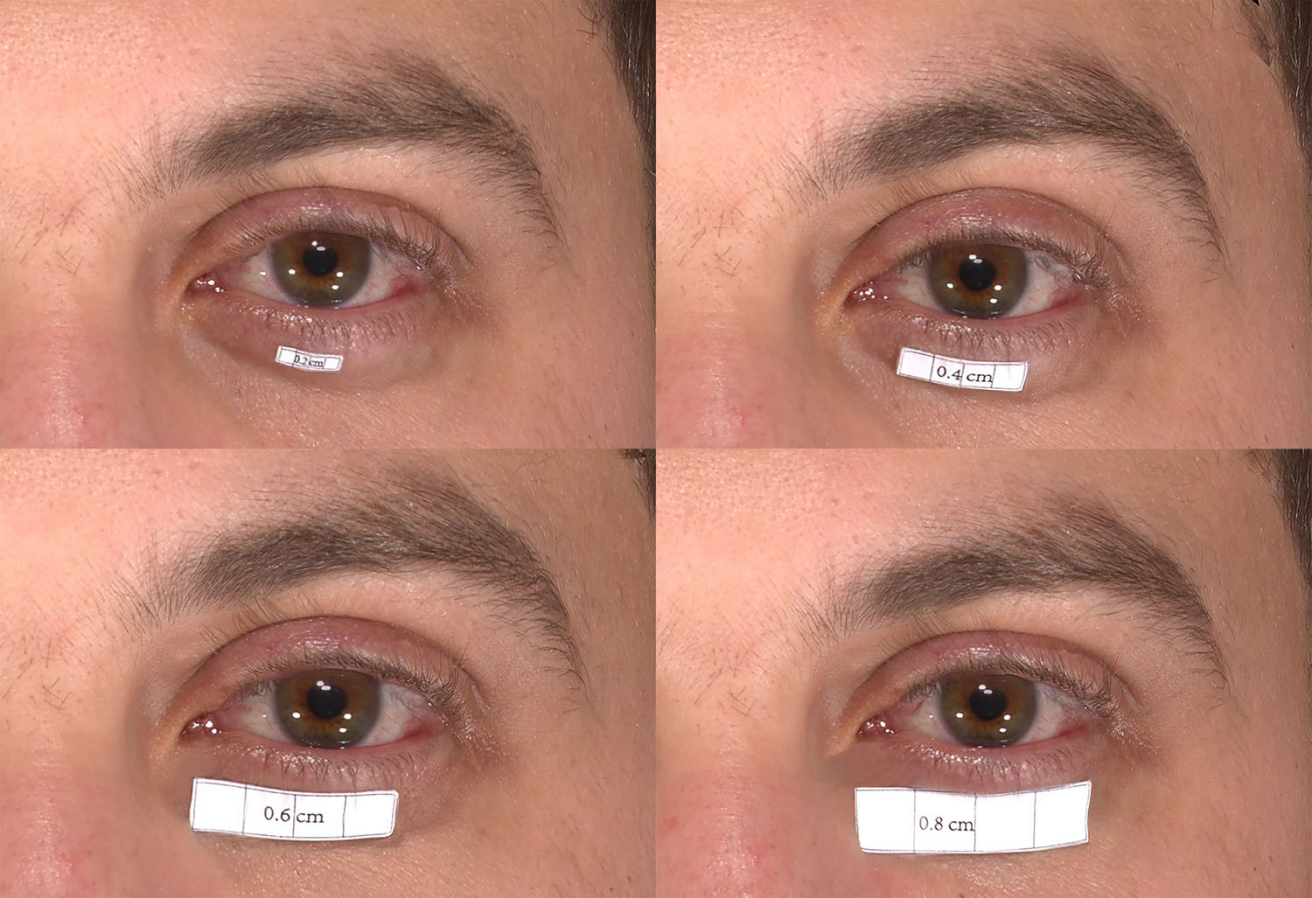
Images of objects 1-4 in the middle of the lower eyelid. Object 1, with an estimated area of approximately 16 mm^2^ (above, left). Object 2, with an estimated area of approximately 64 mm^2^ (above, right). Object 3, with an estimated area of approximately 144 mm^2^ (below, left). Object 4, with an estimated area of approximately 256 mm^2^ (below, right).

**Fig. 2:**
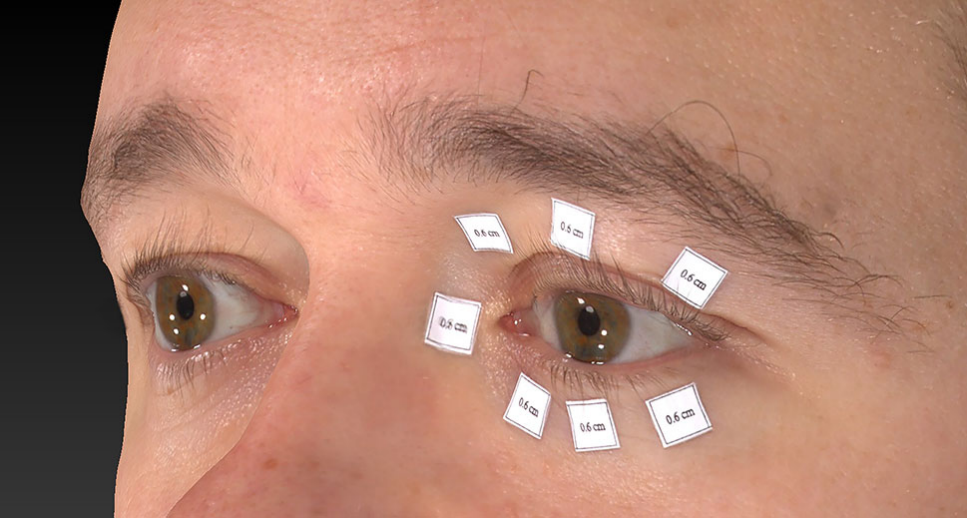
Image of object 5 in seven periocular positions—the endocanthion, and the upper medial, the upper middle, the upper lateral, the lower medial, the lower middle, and the lower lateral.

Five papery objects were designed via Adobe Illustrator 2019 (Adobe Systems, Inc., San Jose, CA, USA). Rectangular-shaped objects numbered 1–4 were designed with an area of 16 mm^2^, 64 mm^2^, 144 mm^2^, and 256 mm^2^, respectively. Square-shaped object 5 was designed with an area of 36 mm^2^. A4 copying paper was used for printing objects. Double-sided adhesive tape with negligible thickness was used to attach the objects firmly to the skin. A caliper was used to measure the size of each object, so as to maintain the area consistency. Objects 1–4 were placed in the middle of the lower eyelid separately beneath the eyelid margin (Fig. 1). Object 5 was pasted in seven different positions—the endocanthion, and the upper medial, the upper middle, the upper lateral, the lower medial, the lower middle, and the lower lateral eyelid (Fig. 2). Each object was kept at a 5 mm distance from the eyelid margin to reduce the impact of eyelashes on measurements.

## Photograph

**Fig. 3:**
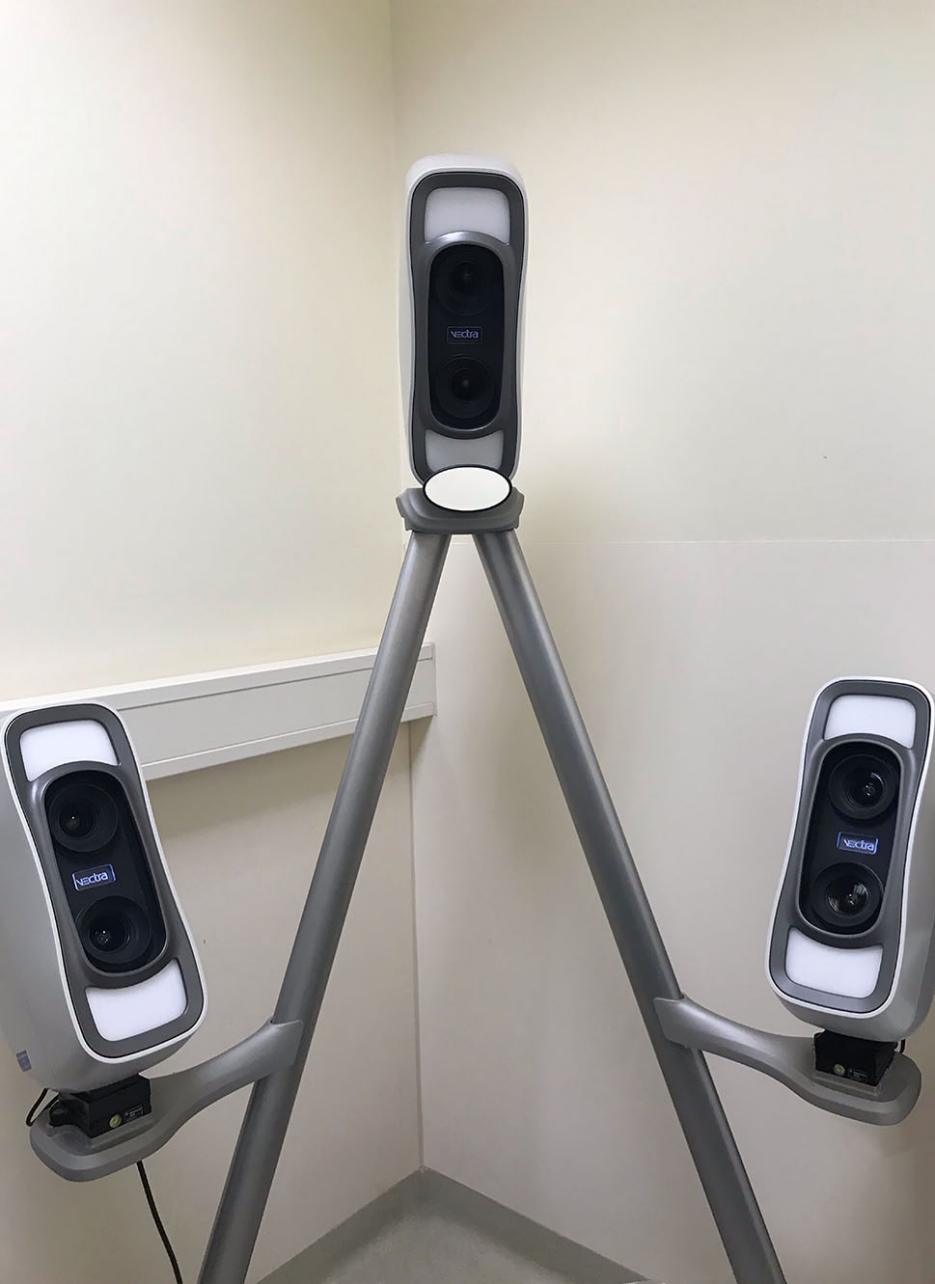
VECTRA M3 system used for Stereophotogrammetry analyses.

Stereophotography was captured with the VECTRA M3 3D imaging system (Canfield Scientific, Inc., Fairfield, NJ, USA), as shown in Fig. 3. Before the photographs were taken, the volunteers’ hairs were pushed back and any jewelry was taken out to prevent facial concealing and abnormal reflections. Facial makeup was removed to achieve a clearer vision of operation. Images were captured while volunteers were sitting in front of the camera with natural expressions in repose. The operating instructions of the camera were exactly followed to gain standard images. Photographic capture was conducted twice by the first author (Series 1 and Series 2). The 3D camera was calibrated before each series.

## Measurements

Each image obtained from Series 1 was measured twice by the rater 1 and rater 2, named as rating 1.1, 1.2 and rating 2.1, 2.2. Images from Series 2 were measured once by rater 2, named as rating 2.3. A time interval of over 24 hours was conducted between measurements. Both of our two raters received systematic and detailed training before starting the measurement to reduce individual differences. Landmarks were placed carefully at the outer edge of each object by the raters when the image was enlarged at a suitable magnification. The size of landmarkers is fixed (radius about 1 mm) by the system and will not be changed as the picture is enlarged. When all the markers are placed in each object, the area enclosed by the line passing through the center of these landmarks was selected as the target area. Area measurement was calculated using the Vectra software (Canfield Scientific, Inc., Fairfield, NJ, USA).

## Data analyses

Five frequently utilized statistical indicators were used to evaluate the reliability of stereophotography, including the intraclass correlation coefficient (ICC), the mean absolute difference (MAD), the technical error of measurement (TEM), the relative error measurement (REM), and the relative TEM (rTEM). The ICC is widely used to evaluate the agreement between measurements. MAD stands for the average absolute value of all the deviations of a single observation from the arithmetic mean. TEM is used in anthropometry to compare the results of measurements between different observers or collection methods. REM and rTEM stand for the estimate of variation relative to the size of the measurements. Guo et al. [11] stated certain calculation formulas for these indicators. Generally speaking, the higher the TEM, REM, rTEM, and MAD values the more significant the discrepancies. However, the lower the ICC value the more significant the discrepancy. From an anthropometry aspect, ICCs < 0.5 are deemed as poor, 0.5 to 0.75 as moderate, 0.75 to 0.9 as good, ICCs > 0.9 as excellent, respectively [14]. While for REM and rTEM, <1%, 1% to 3.9%, 4% to 6.9%, 7% to 9.9%, and ≧10% were considered as excellent, very good, good, moderate, and poor agreement, respectively. In previous studies, the acceptable error threshold for both MAD and TEM was set to beneath two units in the maxillofacial regions, while for the periocular region, some studies believe that it should be less than one unit due to the relatively small magnitude [11].

The intrarater analysis was obtained by comparing rating 1.1 with rating 1.2. The interrater analysis was obtained by comparing rating 1.2 with rating 2.2. The intramethod analysis was obtained by comparing rating 2.2 with rating 2.3.

The bar graphs were generated using GraphPad Prism version 8 (GraphPad Software, Inc., San Diego, CA). All statistical analyses were performed using SPSS software version 22 (IBM Corporation, Armonk, NY). To analyze the differences between the measurements, paired-samples *t*-tests were used for normally distributed data, while nonparametric Wilcoxon signed-rank tests were calculated for non-normally distributed data, and the Kolmogorov-Smirnov test was used to verify normality distribution. The statistical significance level was set at *p* < 0.05.

## Results

Forty healthy young volunteers aged between 18 and 36 years old, including 10 Caucasian males, 10 Caucasian females, 10 Chinese males, and 10 Chinese females were included, with a mean age of 28.1 ± 4.4 years (range 22–38 years). No statistical significance was found between the sexes and races (*p* > 0.05, independent-samples *t*-test).

**Fig. 4:**
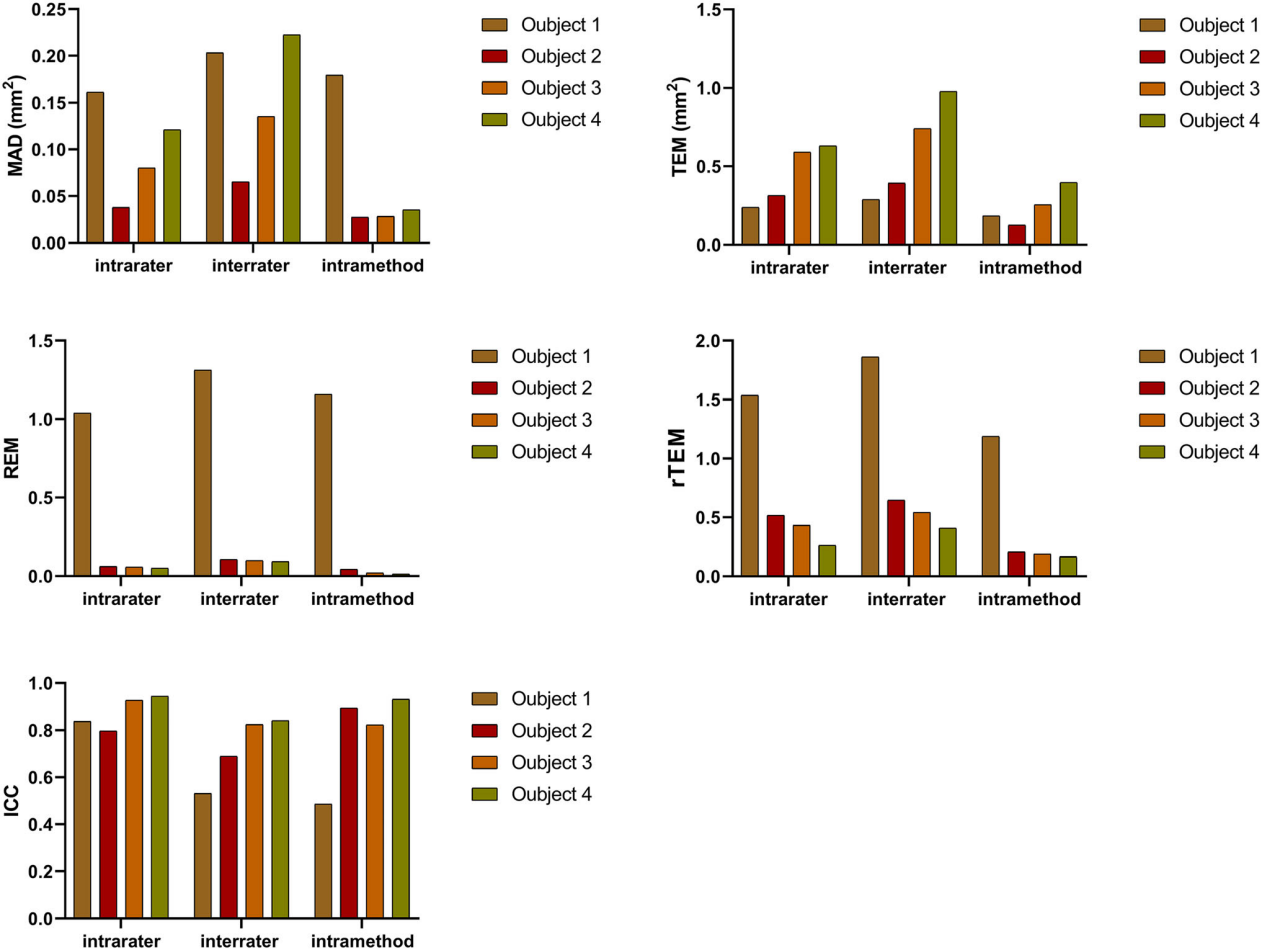
The bar graphs show the intrarater, interrater, and intramethod reliability for the intraclass correlation coefficient (ICC), the mean absolute difference (MAD), the technical error of measurement (TEM), the relative error measurement (REM), and the relative TEM (rTEM) for objects 1-4.

**Fig. 5:**
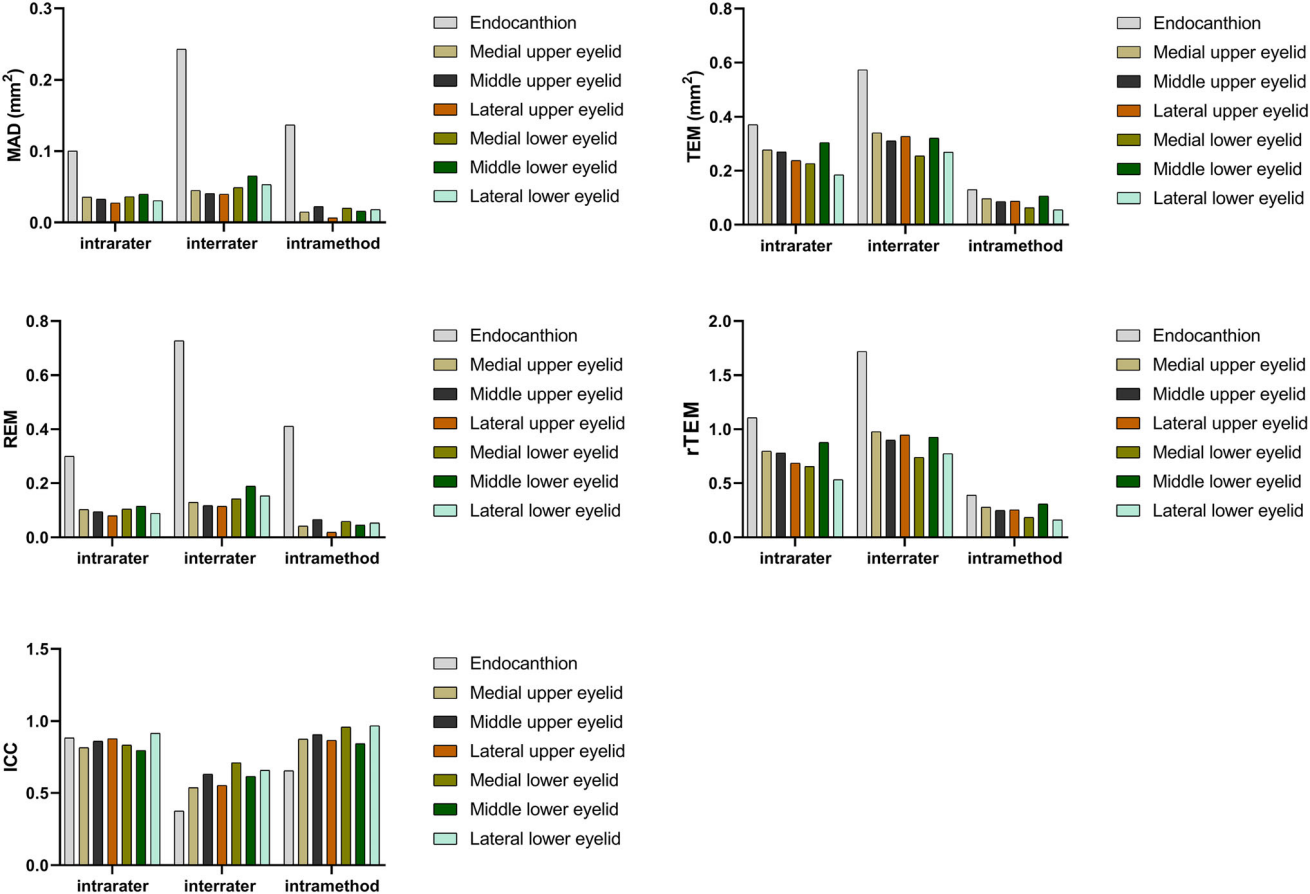
The bar graphs show the intrarater, interrater, and intramethod reliability for the intraclass correlation coefficient (ICC), the mean absolute difference (MAD), the technical error of measurement (TEM), the relative error measurement (REM), and the relative TEM (rTEM) for object 5 in seven different positions.

The differences between measurements using the Wilcoxon signed-rank test are shown in Table 1. No statistical significances were found for the most intrarater, interrater, and intramethod comparations, with exception for the interrater and intramethod for the endocanthion position and the intramethod for the upper middle eyelid position for objects 1 and 5. The intrarater, interrater, and intramethod reliability results for ICC, MAD, TEM, REM, and rTEM for objects 1–4 and object 5 are shown in Figs. 4 and 5, respectively.

**Table 1:** The intrarater, interrater, and intramethod differences between the 3D measurements.

Object	*p* value		
	Intrarater Rating 1.1 versus Rating 1.2	Interrater Rating 1.2 versus Rating 2.2	Intramethod Rating 2.2 versus Rating 2.3
1	0.030^*^	0.020^*^	<0.001^*^
2	0.824	0.253	0.199
3	0.444	0.452	0.468
4	0.532	0.412	0.793
5 (endocanthion)	0.060	0.016^*^	<0.001^*^
5 (upper medial eyelid)	0.638	0.390	0.126
5 (upper middle eyelid)	0.851	0.788	0.027^*^
5 (upper lateral eyelid)	0.677	0.677	0.737
5 (lower medial eyelid)	0.145	0.502	0.153
5 (lower middle eyelid)	0.390	0.307	0.353
5 (lower lateral eyelid)	0.510	0.582	0.185

The *p* value stands for the differences analyzed by the Wilcoxon signed-rank test. *stands for *p* < 0.05

In this study, 21.2% of all ICCs, including the intrarater, interrater, and intramethod ICCs, were considered as excellent and 45.5% of all ICCs were good, 27.3% were moderate, and only the interrater ICC for the endocanthion position and intramethod ICC for object 1 were poor. All intrarater ICCs for objects 1–5 were considered good or excellent, as well as the interrater ICCs for objects 3 and 4. While the interrater ICC for objects 1, 2, and 5 (except for the endocanthion position) were considered as moderate, the interrater ICC for the endocanthion position was deemed as poor. The intramethod ICC for objects 2, 3, 4, and 5 (except the endocanthion position) were considered good or excellent. Meanwhile, the intramethod ICC for object 1 was poor and moderate for object 5 in the endocanthion position. Object 1 possessed the lowest interrater and intramethod ICC of all the objects.

The MAD for all the objects was less than 0.3 mm^2^ despite the different locations. The intrarater MAD for objects 1–4 was between 0.038 and 0.121 mm^2^, while the intrarater MAD for object 5 was less than 0.040 mm^2^. The interrater MAD for objects 1–4 ranged between 0.065 and 0.223 mm^2^, while it was less than 0.066 mm^2^ for object 5. The intramethod MAD for objects 1–4 was between 0.027 and 0.193 mm^2^. The MAD for objects 2–4 increased gradually, while the maximum intrarater MAD and intramethod MAD values were for object 1. The intramethod MAD for object 5 was less than 0.024 mm^2^, except for the endocanthion position, which had a value of 0.137 mm^2^.

The TEM for objects 1–5 was less than 1 mm^2^, to be specific, the intrarater TEM for objects 1–4 was between 0.239 and 0.630 mm^2^, and between 0.185 and 0.371 mm^2^ for object 5. The interrater TEM for objects 1–4 ranged between 0.289 and 0.978 mm^2^, while for object 5 it ranged between 0.255 and 0.573 mm^2^. The intramethod TEM for objects 1–4 was between 0.256 and 0.397 mm^2^, while for object 5, it was between 0.055 and 0.13 mm^2^.

For all the objects, the intrarater, interrater, and intramethod REM was less than 2%. The REM tended to have a low value when the object area increased for all objects. The intrarater REM for objects 1–4 decreased, with a range between 0.1 and 1.0%, while for object 5, it ranged between 0.1 and 0.3%, with the highest value being for the endocanthion position. The interrater REM for objects 1–4 was between 0.1 and 1.3%, while for object 5 it was between 0.1 and 0.8%. The intramethod REM for objects 1–4 was 0% to 1.2% and for object 5 it was 0% to 0.4%.

The rTEM for all the objects was less than 2% and tended to have a similar trend with REM. The intrarater rTEM for objects 1–4 was 0.3% to 1.5% and 0.5% to 1.1% for object 5. The interrater rTEM for objects 1–4 was 0.4% to 1.9%, while for object 5 it was 0.8% to 1.7%. The intramethod rTEM for objects 1–4 was 0.2% to 1.2% and for object 5 it was 0.2% to 0.4%. The endocanthion position possessed the highest value for the intrarater, interrater, and intramethod rTEM for object 5.

The reliability of 20 Chinese was also compared with those from 20 Caucasians. For each same target area, the* p* values of Wilcoxon test were 0.14 (for object 1), 0.270 (for object 2), 0.112 (for object 3), 0.334 (for object 4), 0.570 (for object 5 at the endocanthion), 0.233 (for object 5 at the upper medial), 0.865 (for object 5 at the upper middle and the upper lateral), 0.451 (for object 5 at the lower medial), 0.691 (for object 5 at the lower middle), and 0.191 (for object 5 at the lower lateral). There was no statistically significant difference in reliability between the two different races.

## Discussion

The full application of stereophotography for facial measurements, especially for the periocular area which requires elaborate description, needs to be carefully verified for its technical reliability. Reliability refers to the degree of consistency between repeated measures, also known as precision, repeatability, or reproducibility [3]. Unreliability or imprecision is defined as variability caused by inconsistencies between repeated measurements of the same object and anthropometric aspect [15, 16]. Reliability is a crucial component of validation before a new technology can be applied widely in the clinical setting [17, 18].

This study mainly discusses the reliability of the Vectra M3 stereophotography system while measuring periocular areas with varying objects in different positions, including the reliability of intrarater, interrater, and intramethod, evaluated by five different reliability verification indicators.

In this study, the 3D stereophotography system proved to be highly reliable in most cases. Most of the intrarater and interrater differences were not statistically significant except for object 1 and object 5 in two positions. In this study, 21.2% of all ICCs were considered as excellent and 45.5% of all ICCs were good, 27.3% were moderate, and only the interrater ICC for the endocanthion position and intramethod ICC for object 1 were poor. The MAD for all the objects was less than 0.3 mm^2^ despite the different sizes and locations. All TEMs for objects 1–5 were less than 1 mm^2^. The REM and rTEM for all objects were less than 2% for the intrarater, interrater, and intramethod measurements. In addition, the system showed no statistical difference in reliability when measuring areas from two different races.

Previous studies have shown that the stereophotogrammetry Canfield VECTRA system is a highly reliable novel piece of apparatus for linear and angular measurements [3, 11, 13]. To test the accuracy and reproducibility of the Canfield VECTRA system, de Menezes et al. [19] analyzed the systematic and random errors caused by operators, calibration, and acquisitions and the method was proven to be repeatable. No systematic errors were found, and random errors were less than 1 mm^2^ in their study. A similar result was obtained by Rosati et al. in 2010 for the same system, and no significant differences were found in repeated reproductions [20]. Guo et al. conducted a study in 2019, which supports the high reliability of 3D stereophotogrammetry for periocular linear or angular anthropometry [3]. Their results revealed that, for direct (intrarater reliability only), 2D, and 3D periocular linear or angular anthropometry, intrarater and interrater ICCs were 0.88 for direct anthropometry, 0.99 and 0.97 for 2D anthropometry, and 0.98 and 0.92 for 3D anthropometry; MAD was 0.84 mm for direct anthropometry, 0.26 and 0.36 units for 2D anthropometry, and 0.35 and 0.67 units for 3D anthropometry; TEM estimates were 0.85 mm for direct anthropometry, 0.25 and 0.36 units for 2D anthropometry, and 0.32 and 0.65 units for 3D anthropometry; REM was 6.5% for direct anthropometry, 1.7% and 2.7% for 2D anthropometry, and 1.7% and 5.1% for 3D anthropometry; and rTEM estimates were 6.3% for direct anthropometry, 1.6% and 2.8% for 2D anthropometry, and 2.1% and 5.1% for 3D anthropometry. It can be concluded that, for periocular linear or angular anthropometry, the reliability of 3D measurement is better than direct measurement and comparable to the 2D measurement results. Andrade et al. [21] found that the mean MAD, REM, TEM, and ICC values for nine angular and two linear facial morphology assessments were 1.51 units, 3.6%, 1.35 units, and 0.88, respectively, which shows an excellent agreement in ICC and a very good result for REM. The MAD results were also within the acceptable error threshold. Diana S et al. [22] tested the sagittal projection of six landmarks using the Vectra M5 system in five subjects. Their results proved that the standard deviations for most landmarks were <1 mm^2^ and that mostly the ICC for intrarater and interrater reliability was excellent. Sixteen linear facial measurements were calculated from 10 subjects by de Menezes et al. [19], and the results showed that no systematic errors were found in any of the tests performed (*p* > 0.05). Furthermore, a study performed by Rosati [20] found that the highest mean REM was less than 1.2% in a technical evaluation of the Canfield system in seven linear facial measurements. While in our study, similar results emerged. The REM was less than 1.4% for all objects and 0.8% for objects 2–5.

Although the high levels of geometric reliability of facial linear and angular anthropometry have been validated, studies investigating the reliability of area measurements in the periocular region are rare. Compared with the previously commonly studied maxillofacial region, the periocular structure is more complex and delicate. Thus, the traditional direct measurement and photographic methods are not the optimal choices for taking measurements in the periocular region, compared with the highly repeatable and precise stereophotogrammetry method.

Codari et al. [8] tested the areas of the nasal surfaces, which were measured independently by two operators using the Vectra 3D system, and no significant differences between the operators were found in this study. Daniele et al. [6] validated the repeatability of facial surface area and volume measurements for 50 volunteers via the VECTRA M3 device. Most of the surface area measurements showed high repeatability, with a TEM of 2.70 cm^2^ and an rTEM of 0.8%. In our study, a similar conclusion was obtained with a mean TEM of 3.04 cm^2^ and an rTEM of 0.7% for all objects, although the method Daniele et al. used was different from our study. The facial area of interest was registered and superimposed onto each other to assess the difference in Daniele et al’s. study. In contrast, the area was selected and compared directly to our study. Whether comparing the ICC generated from different methods affects the results is worth further consideration.

In our study, 21.2% of all ICCs were considered as excellent and 45.5% of all ICCs were good, 27.3% were moderate, only the interrater ICC for the endocanthion position and intramethod ICC for object 1 were poor. The discrepancy between the interrater and intermethod ICCs may be related to the differences caused by calibration; besides, it may be associated with different raters drawing different conclusions when faced with fuzzy boundaries. Nevertheless, this does not affect the high ICC reliability for this whole study, which was consistent with the study performed by Guo et al. [11], and it was shown that for the 49 corresponding linear, curvilinear, and angular measurements based on an area within the landmarks, the intrarater ICC was better than the interrater and intramethod ICC.

Interestingly, objects with a larger area tended to produce higher MAD and TEM values in this study; on the other hand, the REM and rTEM values for all the objects decreased as the size of the area increased. This phenomenon has been observed by some studies with linear or angular measurements, in general, a smaller statistical value tends to possess lower MAD and TEM estimates but higher REM estimates [15, 21]. This phenomenon may be caused by the decreasing effect of errors when the relevant magnitude is larger. We could speculate that this phenomenon shows that smaller variables' reliability is more likely to be affected by technical methods and accidental factors.

**Fig. 6:**
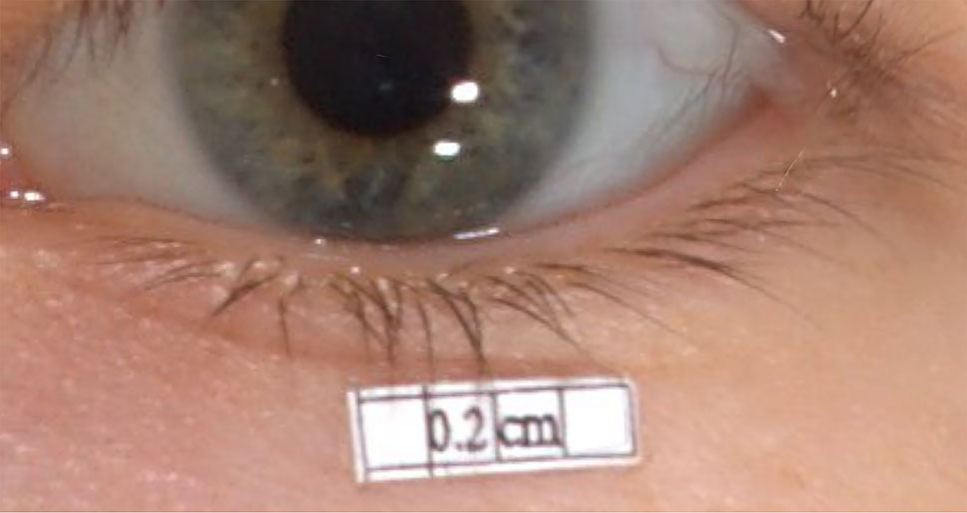
Image of object 1 that shows relatively fuzzy boundaries.

Object 5 in the endocanthion position showed poor reliability among the statistical indicators, similar to object 1. Poor manifestation in the endocanthion position was probably related to the artifacts shown in the images. Among these images, some of the boundaries were not clearly shown, and often appeared as two artifacts. According to our analysis, the occurrence of artifacts may be related to the incomplete development of images due to light occlusion of the nasal or eyebrow bones from the endocanthion. The reason for the low reliability of object 1 may be related to its relatively small target area, as the system's current resolution cannot support more clear boundaries or more accurate measurements, as shown in Fig. 6. Although, the photorealistic rendering of the most exquisite details was improved by using a 36 MB high-resolution image [23].

Although 3D linear and angular measurements have been confirmed by previous studies to be accurate and reliable, interestingly, we still found that the reliability of the endocanthion is inferior compared to other positions in our manuscript, prompting that verifying the areal measurement is still necessary.

In addition, the accuracy is defined as the extent of deviation of a given measurement from its “true” value. Compared with direct measurement and two-dimensional photography, 3D stereophotogrammetry measurement establishes its own three-dimensional coordinate system (horizontally, vertically, and coronally), it reflects a more realistic target location and holds more accurate geometric accuracy. While 2D photogrammetry usually needs to paste a reference object on the position with small variation (such as the forehead), it estimates the measurement value by comparing with the reference object. Also, 2D photogrammetry cannot exclude the influence of depth on target size due to a lack of depth.

In the literature, a clinically acceptable difference between 3D and direct linear measurements was 2 mm or less. Highly accurate was referred to less than 0.5 mm, less accurate, but clinically irrelevant between 0.5 and 1 mm, and clinically relevant between 1 and 2 mm. Dindaroglu et al. conducted a comparion study to evaluate the accuracy of 3D stereophotogrammetry, which focus on to evaluate the accuracy of three-dimensional (3D) stereophotogrammetry (3dMDflex system from 3dMD, Atlanta, Ga) by comparing it with the direct anthropometry (caliper) and digital photogrammetry methods [9]. The results of this study showed that the highest mean difference was 0.30 mm when compared direct measurement to photogrammetry, and 0.21 mm when compared direct measurement to 3D stereophotogrammetry. It suggests that 3D stereophotogrammetry is more accurate in measuring facial variables than 2D photogrammetry.

In our view, for patients whose facial values are within the normal range, 2D photogrammetry may be competitive with 3D stereophotogrammetry. However, for patients whose facial values are not within the normal range (such as those with enophthalmos or exophthalmos), 2D photogrammetry is more likely to get less accurate results than 3D stereophotogrammetry. Besides, 3D stereophotogrammetry is more suitable for diverse facial structures (for example, different races), which has been proved that this technique is stable and reliable when measuring two different races in this study. Also, 3D stereophotogrammetry is more inclusive for head positions when capturing the images, contributing to more convenient operations.

However, 3D stereophotogrammetry system also has its drawbacks. For example, calibration is required before use, and it is unable to capture transparent objects, such as cornea. For some deeper positions, such as endocanthion, it may show less accuracy than other positions due to light blocking, which requires further improvement by the manufacturers. However, it is undeniable that 3D stereophotogrammetry has its unique advantages compared with other measurement methods. When measuring facial variables, it is worthy of further promotion in clinical practice as an accurate and reliable technique.

In general, innovative stereophotogrammetry is becoming a widely used effective evaluation technology for facial differences in a clinical setting [24]. We verified the reliability of area measurements in the periocular region by five frequently utilized statistical indicators. Our study showed high reliability in area measurements despite different positions, sizes and races. To the best of our knowledge, this is the first paper to analyze the reliability of periocular area measurements with stereophotogrammetry systems. This study compiles the data on the reliability of area measurements in the periocular region and may provide a theoretical basis for the relevant variables that need to be assessed, such as surgical selection, the estimated amount of preoperative skin excision, and evaluation for eyelid edema. Furthermore, this study may provide more practical and useful enlightenment for ophthalmologists and plastic surgeons.

Also, this paper has some limitations. Although the sample size is considerable, it is not the largest one compared to similar articles. Besides, as children and elderly people cannot stay still for a long time, their facial structure is different from that of the young. In order not to introduce more variables, our study did not include children and the elderly as research objects.
